# An Efficient Attentional Image Dehazing Deep Network Using Two Color Space (ADMC^2^-net)

**DOI:** 10.3390/s24020687

**Published:** 2024-01-22

**Authors:** Samia Haouassi, Di Wu

**Affiliations:** School of Computer Science and Technology, Dalian University of Technology, Dalian 116024, China; souma@mail.dlut.edu.cn

**Keywords:** image dehazing, HSV color space, pixel-attention, channel-attention

## Abstract

Image dehazing has become a crucial prerequisite for most outdoor computer applications. The majority of existing dehazing models can achieve the haze removal problem. However, they fail to preserve colors and fine details. Addressing this problem, we introduce a novel high-performing attention-based dehazing model ( ${\mathrm{ADMC}}^{2}$ -net)that successfully incorporates both RGB and HSV color spaces to maintain color properties. This model consists of two parallel densely connected sub-models (RGB and HSV) followed by a new efficient attention module. This attention module comprises pixel-attention and channel-attention mechanisms to get more haze-relevant features. Experimental results analyses can validate that our proposed model ( ${\mathrm{ADMC}}^{2}$ -net) can achieve superior results on synthetic and real-world datasets and outperform most of state-of-the-art methods.

## 1. Introduction

Image dehazing is a core concept in digital image processing, the process of eliminating haze from images. It has become a crucial task in the field of computer vision due to its extensive applications in areas such as maritime and air transport, driver assistance systems, surveillance, and remote sensing. The challenge of dehazing lies in the fact that haze is an atmospheric phenomenon that can substantially degrade the quality of images, obscuring details and reducing contrast, color fidelity, and visibility.

In the literature, researchers first have used image enhancement based methods to enhance image intrinsic properties (such as contrast, brightness) and details. In [1,2,3], authors have employed common contrast enhancement techniques to enhance the visibility of hazy images. However, these techniques can only handle hazy images with thin haze and would lead to some distortions and halo effects in case of thick haze. Also, the Retinex theory has been exploited to remove haze from single images [4,5] by enhancing the brightness of the image. This technique can remove the haze and enhance the vividness of colors, but it cannot be able to preserve edge properties.

Differently, using fusion-based strategy, Schechner et al. [6] and Liang et al. [7] have combined the visible light image and the near-infrared image to obtain a high-quality image free from haze. These methods also has attained satisfactory results, however, since additional information is required, the restoration would be a little bit complicated, because a single hazy image cannot provide easily much useful information.

Recently, the image restoration-based Dehazing strategy is extensively studied considering the image degradation source. With the appearance of this scattering degradation model, many researchers have built their Dehazing solutions on it, to recover the scene radiance of degraded hazy images. It can be expressed as follows:

(1) $$I\left(x\right)=J\left(x\right)\tilde{t}\left(x\right)+A\left(1-\tilde{t}\left(x\right)\right).$$

where I(x) is the captured hazy image, J(x) is the scene radiance to be recovered. A(x) represents the atmospheric light and $\tilde{t}$ (x) denotes the transmission medium. x symbolizes image pixels.

**Figure 1:**
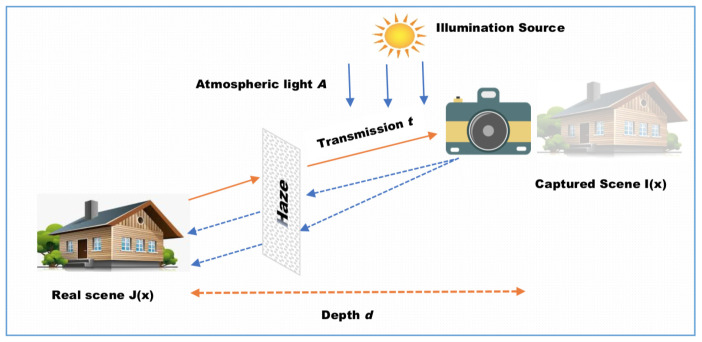
Hazy Image Degradation Model.

Figure 1 describes how haze affects captured images.

This model requires the estimation of two crucial keys, transmission map $\tilde{t}$ (x) and the atmospheric light A to perform the Dehazing process.

Accordinly, He et al. [8] have proposed the dark channel prior (DCP) assumption that sourced from remote sensing of natural haze-free images. Its principle is that for most patches in clear outdoor images, at least one color channel contains very low intensity for some pixels excluding sky region. This assumption can effectively handle thick-haze images and provide the transmission map simply. Despite that, it has significant block effects and halos on the transmission map of the sky region. On the other hand, it is computationally expensive because of the soft matting used in the refinement. Plenty of image dehazing techniques have been proposed [9,10,11]. Berman et al. [9] have proposed haze-lines based-dehazing method to restore the scene radiance by estimating the transmission map distance. Nevertheless, this method suffers from significant problems, such as color distortion and fails for thick-haze images. In contrast, Fan et al. [10] have proposed a dehazing method based on a fusion between a visible image and the near-infrared image. Despite, the pleasant achievement of this method, it has divers halo artefacts may be seen, and it is not easy to get the source images. Based on the fact that the haze thickness is related to the blurriness of the hazy image, Haouassi et al. [11] have presented a powerful Dehazing algorithm that produces excellent results, even so, it doesn’t work with nighttime hazy images.

With the success of machine learning in many image processing related fields, numerous deep learning-based Dehazing methods [12,13,14,15] have been proposed to achieve better Dehazing results and restore clear images with high visual quality. Such Dehazing methods perform well on both synthetic and outdoor hazy images. An End-to-End framework called “DehazeNet” has been presented in [12] that adopts the Convolutional Neural Networks (CNN) algorithm. It takes as input a single hazy image and gives the estimated transmission map as output, and according to the experimental results, it attains high performance. In [13], Golts et al. have designed a different dehazing network using real-world outdoor images and minimizing the Dark Channel Prior (DCP) energy function. Song et al. [14] proposed an efficient Dehazing method based on Ranking-CNN model, which facilitates the haze-relevant feature extraction process. This proposed approach shows its high performance through extensive experimental results on both synthetic and real-world hazy images. However, it is computationally expensive because of the ranking layer.

In our previous paper [15], we proposed an efficient Dehazing algorithm based on the estimation of both key components, transmission map and the atmospheric light. The transmission map is generated automatically by a cascaded Multi-scale CNN termed as (CMTnet), and the atmospheric light value is estimated using an effective algorithm called A-Est. This method can produce high-quality free-haze images but it ability to preserve colors is poor.

So far, the majority of proposed Dehazing algorithms are suffering from non-preservation of real-scene colors and fine details or unsatisfying results (for instance: halo effects in specific regions, over-enhancement). Each of these proposed methods has its level of success in haze removal process.

In summary, we can present our contributions on several points:

- To retain color properties (Saturation, brightness), we integrate the HSV color space and RGB color space rather than RGB only.
- We propose an efficient attention module that incorporates both channel-level and pixel-level to ensure improved color preservation and varying haze conditions adaptability. This attention mechanism is powered by mixed-pooling technique (Fusion of Max pooling and Average pooling) that can be beneficial to improve the performance and robustness of the model.
- To generalize well the proposed model we train it with both synthetic data and real-world challenging benchmarks (Reside [16], O-haze [17], Dense-haze [18], and NH-haze [19]).

## 2. Related Works

Single image Dehazing has become a topic of interest in recent years, due to its great importance in many computer vision-related fields. The traditional dehazing techniques predominantly relied on image processing, involving the direct manipulation of pixels or the utilization of manually crafted priors [1,2,3,4,5,6,7,8,9,10,11].

### 2.1. Prior-Based Dehazing Techniques

These methods employ diverse prior information or assumptions related to the scene, haze distribution, or the image itself to estimate and remove the haze, such as DCP [8], haze-line model [9], color attenuation prior [20], and Non-local Color Prior (NCP) [21]. He et al. [8] introduced the dark channel prior, positing that the minimum intensity within the RGB channels at a local level should approximate zero in haze-free natural images. DCP pixel tends to be associated with a non-hazy scene. It is used to estimate the transmission map, which represents the scene’s haze. In [9], a Haze-Line based dehazing model is proposed, it operates on the premise that the color of a scene undergoes linear changes in relation to depth. This model employs this assumption as a basis for estimating the transmission map, allowing for the effective removal of haze from the image. Also, Zhu et al. [20] have introduced a Color Attenuation Prior method that operates under the assumption that the color of a scene exhibits greater consistency in the transmission-reflection model in HSV color space. In [21] the Non-local Color Prior (NCP) is introduced, leveraging the insight that a crisp image is characterized by a limited number of clusters within the RGB color space.

Although these prior-based methods have proven effective in many scenarios, they are not applicable in complex hazy scenes.

### 2.2. Deep Learning-Based Dehazing Methods

These techniques have carried about a revolutionary shift in the field of image dehazing, significantly improving performance over traditional techniques.

Unlike prior-based approaches that rely on evident assumptions or handcrafted priors, deep learning models, in particular convolutional neural networks (CNNs), can adapt and generalize across various landscapes. Moreover, deep learning models can easily handle noise and learn scene-related features contributes to enhanced dehazing performance.

In [12] Cai et al. have proposed an end-to-end trainable CNN model that can generate transmission map for the input hazy image, which is subsequently used to recover a clear image via the atmospheric scattering model. This model has shown superior dehazing results comparing with traditional methods, however, it can not fully capture the diverse range of real-world hazy conditions. Likewise, MSCNN [22] introduced a dehazing approach that progresses from coarse to fine by establishing a multi-scale convolution network. This model shows promising results in haze removal however it may suffer from constructive errors. Differently, Li et al. [23] have introduced an All-in-One model that employs a reformulated Equation (1) to estimate both transmission medium and airlight simultaneously. Many studies [14,15,24,25] have employed deep learning-based technologies and show promising dehazing results, however, cannot handle complex senes and varying haze conditions scenes. Additionally, most of these proposed deep-learning based dehazing techniques may suffer from computational complexity because of the feature redundancy, which leads to inaccurate dehazing outcomes particulary in challenging scenes (non-uniform haze, variying light conditions,..., etc.).

To address these issues, several researchers have joined the attention mechanism with deep learning techniques in image dehazing task, to selectively emphasize or de-emphasize certain parts of the input hazy image.

### 2.3. Attention Mechanisms-Based Deep Learning Dehazing Methods

Recently, the integration of attention mechanisms into deep learning models for image dehazing tasks has been pivotal for optimizing the model’s performance. Plenty of works have adopted attention mechanisms to improve the effectiveness of dehazing-based models. In [26], the authors have presented a robust attention-based multi-scale network that consists of three key components and implements a novel effective attention mechanism to capture haze-relevant features. It has been demonstrated to promote good results; however, it has some inherent limitations, such as sensitivity to lighting conditions, and poor performance on thick haze scenes. Also, in [27,28] authors have proposed pixel-channel attention models to address the non-uniform distribution of haze at different pixel levels. CP-net [27] incorporates a double attention module (DA), and FFA-net [28] introduces a powerful feature attention module along a basic residual block. Furthermore, an effective network was introduced in [29] it comprises a residual spatial and channel attention module to adaptively adjust feature weights, considering haze distribution, enhancing feature representation and dehazing performance. Moreover, Sun et al. [30] have proposed a fast and robust semi-supervised dehazing method (SADnet) that incorporates both channel and spatial attention mechanisms. This technique shows its effectiveness in haze removal; however, it produces some color artefacts on dehazing outcomes.

It is evident that the above-discussed attention-based models [26,27,28,29,30] can exhibit notable improvements in enhancing dehazing robustness. They outperform existing end-to-end models, showcasing their efficacy in addressing haze-related challenges. However, they can produce some color artefacts and cannot handle complex real-world scenarios. Addressing these challenges and further refining the existing methodologies will be pivotal in advancing the field and ensuring the practicality of these models in real-world settings, with different lighting conditions and degrees of complexity.

To address such weaknesses, this paper leverages the synergistic advantages benifiting from the fusion of channel and pixel attention, along with the use of various color spaces (RGB and HSV). The fusion of channel and pixel attention allows the model to refine its focus localy and globally, enabling a more precise and context-aware dehazing process. Moreover, the incorporation of two color spaces further enriches the feature representation, exploiting characteristics of each space. By integrating information from RGB, HSV color representations, the model enhances its ability to deal with complex color variations in hazy scenes, resulting in superior color restoration.

This multi-attentional and multi-colour space strategy contributes to the robustness and adaptability of the proposed method, making it well-suited for a broad spectrum of real-world dehazing scenarios. The proposed network ( ${\mathrm{ADMC}}^{2}$ -net) details are discussed in the next section.

## 3. Proposed Learning-Based Image Dehazing Method

**Figure 2:**
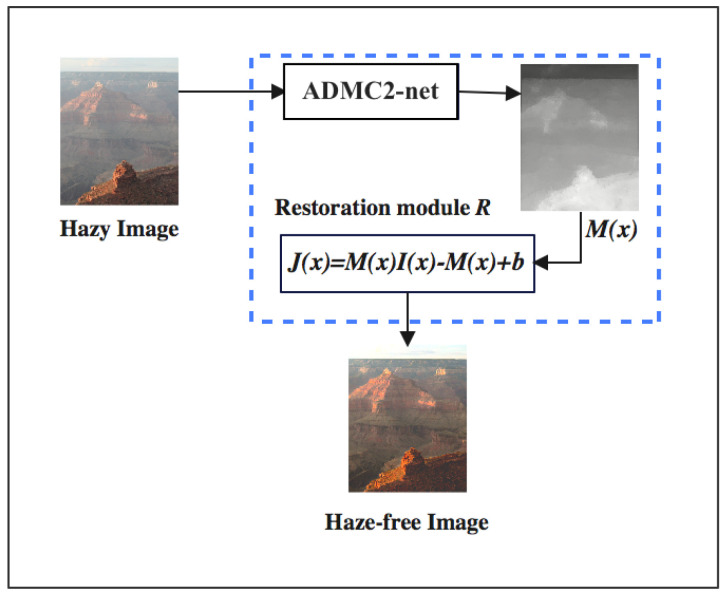
Overall design of the proposed end−to−end model.

In this section, we discuss the detailed framework of the proposed Dehazing network ( ${\mathrm{ADMC}}^{2}$ -net). The proposed model consists of a two-path block of trained dense units (D-Unit), a concatenation module, a Channel-Pixel fusion attention module, and a restoration module. First, we provide an overview of the proposed Dehazing network Figure 2. Second, we present the network’s main components in detail, including color space used, dense units (D-unit), concatenation, channel and pixel modules, fusion attention, and restoration module. Then, we introduce the loss function to train the network.

### 3.1. Overview of the Proposed Architecture

Inspired by the impressive success of end-to-end deep neural networks in image dehazing field [12,23], we propose a robust dehazing model ( ${\mathrm{ADMC}}^{2}$ -net), that can effectively generate the mapping M(x) directly and minimize the reconstruction errors.

Our network ( ${\mathrm{ADMC}}^{2}$ -net) inspires AOD-Net’s [23] overall design as shown in Figure 2 to generate clear images from hazy ones directly.

**Figure 3:**
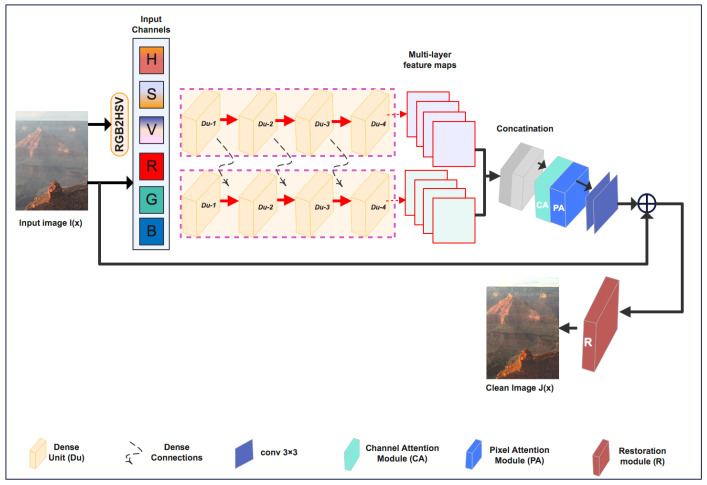
Architecture of the proposed dehazing model ( ${\mathrm{ADMC}}^{2}$ -net).

As shown in Figure 3, the input hazy image undergoes an RGB to HSV colorspace conversion. Then, we create two paths, RGB and HSV, to extract more informative features separately. After that, the input feature maps were fed into four consecutive dense units (D-units) in each path, obtaining different representative and informative feature maps from the two parallel paths, RGB and HSV.

Densely connecting the RGB path’s features with the HSV path’s features can improve the performance of the network D-unit block. The generated RGB and HSV features are then input into a recovery module after being fed into a fusion attention module to extract the most important features.

Our proposed dehazing network ( ${\mathrm{ADMC}}^{2}$ -net) can handle the image dehazing issue effectively in terms of image quality and haze removal.

### 3.2. Network Architecture

Our proposed model ( ${\mathrm{ADMC}}^{2}$ -net) consists of four key components: HSV-RGB Color Space Representation, dense units (D-unit), Fusion Attention Module, and Recovery Module. In this section, we will explain each of these components in more detail.

#### 3.2.1. Two-Color Space Model

Generally, most of proposed dehazing methods process in the RGB color space, it has a strong physical color property that allows for easy image display and storage; However, its channels have a high level of correlation, so the entire image’s appearance might be affected easily through luminance changes, lights, shadows, white areas, haze drops, and other factors. As we mentioned before, the majority of recent image dehazing techniques have the ability to remove the haze from single hazy images in most cases, however; these methods have shown their sensibility to lighting conditions and produced some color artefacts on the dehazing outcomes.

Our proposed dehazing model takes a step further by incorporating feature extraction from two color spaces, RGB and HSV. This unique combination offers significant benefits for image dehazing, as it enables better representation of visual information and enhances the ability to handle both color distortions and halo artifact challenges. By leveraging the strengths of both color spaces, our model delivers superior results compared to traditional dehazing techniques.

RGB is an additive color model widely used in electronic displays, such as computer monitors, television screens, and digital cameras. It is composed of three color channels, Red, Green, and Blue, each channel rangesfrom 0 to 255 (8 bits per channel).

HSV is a cylindrical model where the hue is the angle of the color relative to red, the saturation is the intensity of the color relative to the hue, and the value is the brightness of the color. HSV is often preferred for color representation and manipulation as it is more intuitive and easier to work with. It consists of three independent attributes: hue (H), saturation (S), and value (V). Hue specifies the property of color, saturation represents the purity of a specific color, and value signifies pixel intensity and brightness.

- Hue (H): 0 to 360 degrees.
- Saturation (S): 0 to 1
- Value (V): 0 to 1

Furthermore, the HSV color space has proven to be highly effective in a variety of image-related tasks [31,32,33,34], including image deahzing task, yielding exceptional results. This benefit has motivated us to combine the advantages of both the HSV and RGB color spaces.

HSV-RGB color space integration solves most learning-based dehazing problems, color costs, and halo artifacts. HSV promotes brightness and color, whereas RGB enhances detail. A dehazing network using both color spaces increases data-driven learning and robustness. Later layers may focus on intensity-related variables as the model learns to blend HSV and RGB properties. Real-world applications with varied input images need this generalization.

Network funtionality: The input of our network is a single hazy image.

RGB−network: First, RGB channels are fed into a four D-units sub-network with multiple skip connections. Denote $I_{RGB}$ is the input hazy image with three channels {R,G,B }, and $W_{i}$ , $b_{i}$ represent the weights and biases of the i-th D-units, respectively. *f* is the activation function. The output of each D-unit can be expressed as:

(2) $$Y_{1}=f\left(W_{1}\cdot I_{RGB}+b_{1}\right)$$

(3) $$Y_{2}=f\left(W_{1}\cdot Y_{1}+b_{2}\right)$$

(4) $$Y_{3}=f\left(W_{1}\cdot Y_{2}+b_{3}\right)$$

(5) $$Y_{4}=f\left(W_{1}\cdot Y_{3}+b_{4}\right)$$

Then, the overall network expression can be expressed as:

(6) $$Y_{4}=f\left(W_{4}\cdot \left(f\left(W_{3}\cdot \left(f\left(W_{2}\cdot \left(f\left(W_{1}\cdot I_{RGB}+b_{1}\right)\right)+b_{2}\right)\right)+b_{3}\right)\right)+b_{4}\right)$$

HSV−network: First, the input hazy image is transformed to HSV representation (RGB—HSV), it is used as an input for the HSV subnetwork. Then, the process is quite similar to what we explained for RGB image. However, HSV image typically has three channels (H,S,V), so the dimensions of matrices weights must be ajusted.

The final network formula can be expressed as:

(7) $$Z_{4}=f\left(W_{4}\cdot \left(f\left(W_{3}\cdot \left(f\left(W_{2}\cdot \left(f\left(W_{1}\cdot I_{HSV}+b_{1}\right)\right)+b_{2}\right)\right)+b_{3}\right)\right)+b_{4}\right)$$

where $I_{HSV}$ = h,s,v where H, S, and V are the individual channels. $W_{i}$ , $b_{i}$ represent the weights and biases of the i-th D-units, respectively. f is the activation function.

Finally, the output features $Y_{4}$ and $Z_{4}$ are fused and concatenated with the HSV-network’s features. Finally, all these features passed through the proposed feature attention module. In the next subsections we give details of D-unit and Feature Attention Module.

#### 3.2.2. D-Unit Structure

**Figure 4:**
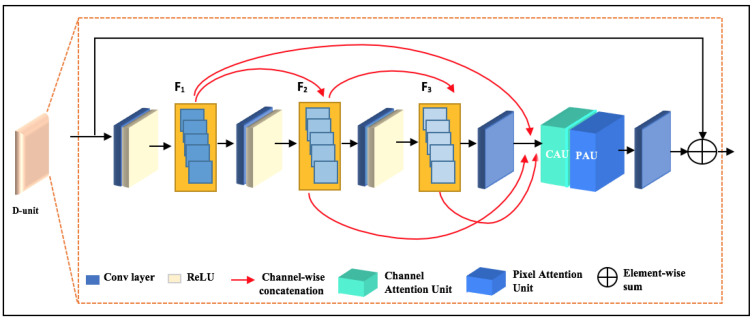
Structure of D-unit.

The D-unit consists of dense convolutions network and a feature attention module. Inspired by our previous work [15], we exploit the D-unit architecture to build the two-color space dehazing model ( ${\mathrm{ADMC}}^{2}$ -net). Figure 4 shows the general internal structure of a D-unit. To reduce the computational complexity while also increasing the receptive field, we use (3 × 3) convolutions followed by a rectified linear unit (ReLU) (except for the last convolutional layer). The DenseNet [35] framework inspired the basic concept of a D-unit, in which each convolutional layer is connected to all other layers in a feed-forward manner. For instance, feature maps of the first layer C1 are densely connected to all subsequent layers C2, C3, and C4.

Most traditional deep learning-based dehazing methods treat pixel-wise and channel-wise features equally. As a result, these methods cannot deal with dense haze and uneven haze distribution scenes. In contrast, Qin et al. [28] proposed a robust feature attention mechanism that can be more flexible with different hazy scene types and treats all haze levels well. It comprises channel attention and pixel attention, as explained in the next section.

The proposed unit architecture has many benefits that make it more advantageous than other deep learning-based dehazing models. These benefits includes its ability to avoid the gradient vanishing problem that deep CNNs have. In addition, this architecture is designed to maximize the flow of information with non-redundancy of feature maps. This proposed unit can highlight dense haze and bypass the less important information like low frequency regions and thin haze, which can lead to outstanding haze removal results.

#### 3.2.3. Feature Attention Module

Recently, the attention mechanism has become increasingly crucial in deep learning-based dehazing methods. It shows an outstanding ability to help the model adaptively process the most critical parts while ignoring irrelevant ones.

Several studies [28,29,30] have employed this mechanism to enhance the effectiveness of DNN-based dehazing models by incorporating channel attention, pixel attention or both. However, these attempts failed to yield satisfactory results because of the difficulty of tackling intense haze in scenarios with dense haze, the complexity for integration to other existing DNN-based dehazing methods, and limited robustness due to sensitivity to some variations, such as scene complexity, lightning conditions, or weather.

By addressing these problems, we propose an efficient and robust attention module that inspires the significant achievement of fusing channel and pixel mechanisms for image dehazing task.

**Figure 5:**
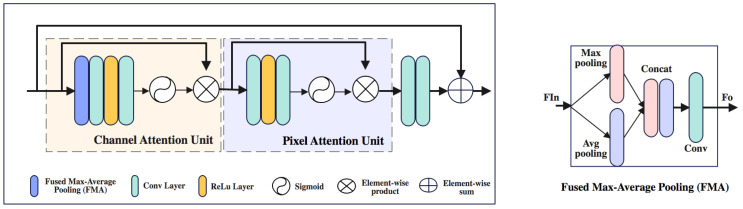
Sheme of proposed Attention module.

As depicted in Figure 5, our proposed attention module inspires FFA-Net [28] attention architecture, which employs w fusion of both channel and pixel attention mechanisms. However, for channel attention stage we employ a hybrid max-average pooling technique that can capture a more comprehensive representation of the input by preserving global and local informatioin within the model.

First, for channel attention we use max-average (MA) pooling to transform channel-wise global spatial information into a channel descreptor.

(8) $$MA=F_{cat}\left(P_{max}\left\{F_{in}\right\},P_{average}\left\{F_{in}\right\}\right).$$

Then, the features undergo two convolutional layers, with ReLU activation function λ and then a sigmoid function σ .

(9) $$C_{c}=\sigma \left(conv\left(\lambda \left(conv\left(MA\right)\right)\right)\right)$$

Finally, we perform element-wise multiplication between the input feature $F_{in}$ and the channel weights $C_{c}$ .

(10) $$F_{c}=C_{c}⊗F_{in}$$

Similarly, we input $F_{c}$ (output of channel attention) to two convolutional layers with ReLu λ and the sigmoid activation function σ .

(11) $$PA_{c}=\sigma \left(conv\left(\lambda \left(conv\left(F_{c}\right)\right)\right)\right)$$

In the end, multiplying the output $PA_{c}$ and the input $F_{c}$ element-wise.

(12) $$F_{att}=PA_{c}.⊗FC$$

This feature attention module adopts a lightweight architecture that can be smoothly fused into existing image-dehazing models without imposing significant computational costs. It aims improve the performance of image dehazing model by effectively augmenting the overall clarity and quality of the dehazed results and preserving varying haze conditions adaptability.

Overall, the proposed attention model demonstrates its ability in dealing with most of dehazing challenging problems in particuraly, varying lightening and vaze conditions, color distortion, contextual information preservation.

#### 3.2.4. Loss Function

Generally, image dehazing models employed L2 loss MSE (Mean Squared Error) to train the model efficiently because of its simplicity and ease of converging to an optimal solution. However, it has limitations regarding overly smoothed outputs that can not preserve textures or details well. Its main goal is to minimize the error between output and the ground truth of a hazy image. MSE loss is expressed below.

(13) $$L_{MSE}=\frac{1}{M}\sum_{i=1}^{M}{\left(I_{gt}-I_{pred}\right)}^{2}$$

where $I_{g}$ is the ground-truth image and $I_{pred}$ represents the predicted output, *M* is the number empirical values.

As we mentioned above, MSE is a pixel-wise metric that can not maintain the perceptual quality of an image and can produce overly smoothed mages. In contrast, a robust dehazing system ought to not only effectively remove haze but also preserve edges, textures, and fine details.

On the other hand, SSIM (Structural Similarity Index) encourages structural details preservation. It considers three key components to evaluate the perceptual similarity between two images: structure, contrast, and brightness. Its value varies between −1 and 1, where 1 represents an excellent similarity.

(14) $$L_{SSIM}=1-\frac{1}{M}\sum_{p=1}^{M}\left(\frac{2\mu_{x}\mu_{y}+C_{a}}{\mu_{x}^{2}+\mu_{y}^{2}+C_{b}}\cdot \frac{2\sigma_{xy}+C_{a}}{\sigma_{x}^{2}+\sigma_{y}^{2}+C_{b}}\right)$$

where $C_{a}$ and $C_{b}$ represent constants of regularization, *x* and *y* are the compared images. $\mu_{x}$ and $\mu_{y}$ are averages of *x* and *y*, and $\sigma_{x}^{2}$ , $\sigma_{y}^{2}$ represent variances of *x* and *y*.

For image dehazing, choosing the loss function requires a trade-off between pixel accuracy and perceptual feature preservation. Therefore, combining SSIM loss and MSE loss can be beneficial for training and help the dehazing model handle all distortions and challenging scene conditions.

In this research we employ a combination of L2 loss ( MSE ) and SSIM loss, which can be defined as follows.

(15) $$L_{t}=L_{MSE}+L_{SSIM}$$

## 4. Results

**Figure 6:**
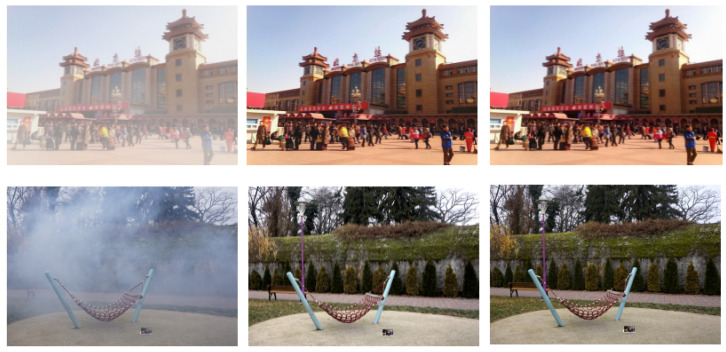
Some Visual Results of Proposed Dehazing Model: The hazy image, our result, Ground truth, respectively.

In this section, we disscuss the dataset and experimental setup used in our study (some visual results Figure 6). Furthermore, the proposed dehazing model is first assessed by conducting various experiments using state-of-the-art dehazing methods on real-world and synthetic data.

The validation of these investigations incorporates both qualitative visual effects and quantitative assessment metrics. Finally, we perform ablation studys to prove the significance of each unit in our proposed architecture.

### 4.1. Datasets and Implementation

#### 4.1.1. Datasets

To assess the effectiveness of our innovative dehazing model, we performed comprehensive experiments on real-world images and synthetic datasets. For our experiments, we chose the publicly accessible large-scale dataset RESIDE (REalistic Single Image DEhazing) [16]. It contains thousands of pairs of indoor and outdoor hazy images as well as their corresponding ground truth.

For training, we picked 2500 synthetic hazy and corresponding ground truth images from OTS (Outdoor Training Set) and 500 from ITS (Indoor Training Set). For validation on synthetic and real-world images, we select 400 synthetic hazy images from SOTS (Synthetic Objective Testing Set) and 15 real-world foggy images from HSTS (Hybrid Sujective Testing Set).

Furthermore, we trained our approach on real-world challenging haze-removal benchmarks, Dense-Haze [18], NH-HAZE [19], and O-HAZE [17] datasets, 90% used for training and 10% for evaluation.

#### 4.1.2. Implementation Details

In our experiments, for training, we utilized an ADAM optimizer as an optimization approach to update network parameters recurrently. The model was trained using the standard learning rate of 0.001 and network parameters $\beta_{1}$ and $\beta_{2}$ set to 0.9 and 0.999, respectively, with 200 epochs. We implement our network on a computer of 12th Gen Intel(R) Core i5-12600k, GPU is NVIDIA RTX 2080Ti.

### 4.2. Comparisons and Experimental Results

In this section, we perform an experimental analysis by comparing our proposed model to other existing approaches, both traditional and learning-based, TA-3DP [36], MB-TF [37], GRIDdehaze-Net [26], CMTnet [15], GEN-ADV [38], DP-IPN [25], ADE-CGAN [39]. First, we conduct a qualitative comparison to assess the compared methods on both synthetic and real-world hazy images. Then, to confirm the qualitative comparison analysis, we perform a quantitative comparison using the extensively used metrics SSIM and PSNR, and $\Delta E_{ab}^{*}$ [40] that can compare two images in terms of color, it is a color difference function.

#### 4.2.1. Qualitative Comparison on Both Real-World and Synthetic Hazy Images

**Figure 7:**
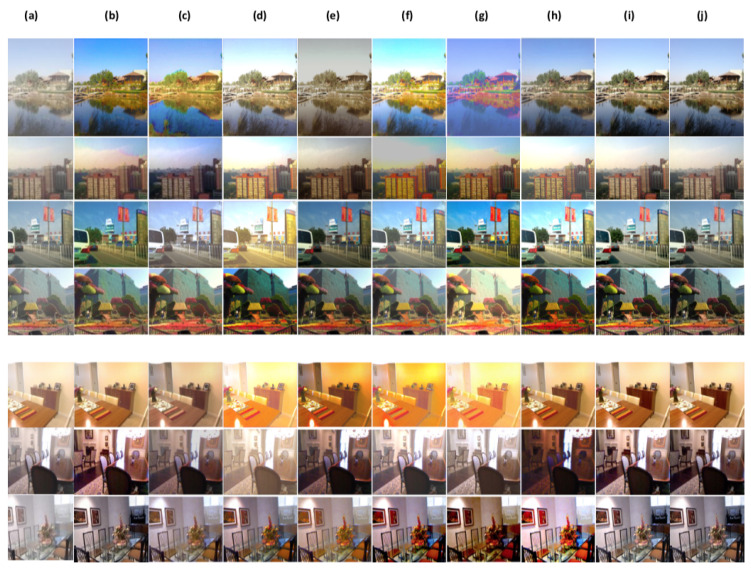
Subjective comparison on synthetic dataset RESIDE. (**a**) hazy image, (**b**) TA-3DP [36], (**c**) MB-TF [37], (**d**) GRIDdehaze-Net [26], (**e**) CMTnet [15], (**f**) GEN-ADV [38], (**g**) DP-IPN [25], (**h**) ADE-CGAN [39], (**i**) Ours. (**j**) Ground truth.

**Figure 8:**
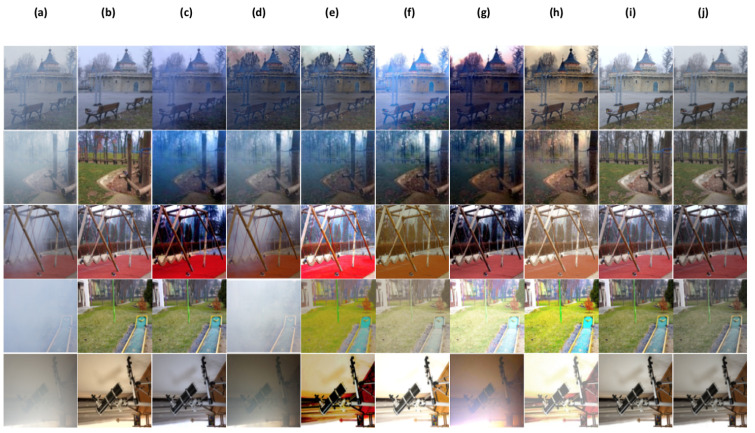
Subjective comparison on Real-world datasets Dense-Haze, NH-HAZE, and O-HAZE. (**a**) hazy image, (**b**) TA-3DP [36], (**c**) MB-TF [37], (**d**) GRIDdehaze-Net [26], (**e**) CMTnet [15], (**f**) GEN-ADV [38], (**g**) DP-IPN [25], (**h**) ADE-CGAN [39], (**i**) Ours. (**j**) Ground truth.

**Figure 9:**
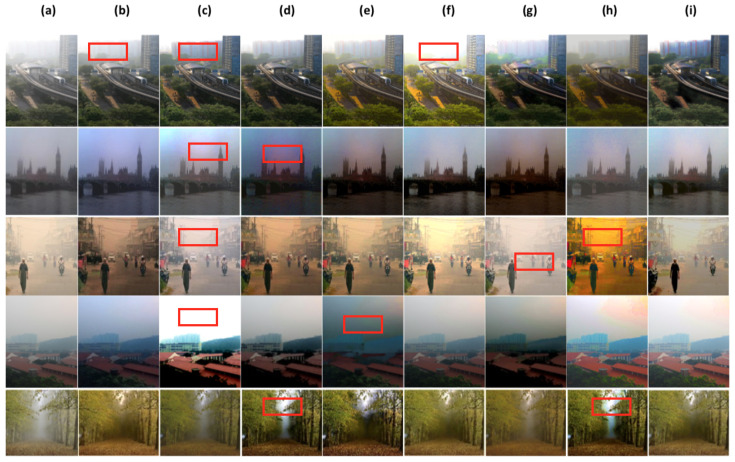
Some Visual comparisons of comparing methods on Real-world images (without ground truth). (**a**) hazy image, (**b**) TA-3DP [36], (**c**) MB-TF [37], (**d**) GRIDdehaze-Net [26], (**e**) CMTnet [15], (**f**) GEN-ADV [38], (**g**) DP-IPN [25], (**h**) ADE-CGAN [39], (**i**) Ours.

In this part, we conduct a qualitative comparative analysis, by presenting a range of dehazing results for the above methods [15,25,26,36,37,38,39] including ours. In Figure 7, Figure 8 and Figure 9, we illustrate outcomes obtained from both synthetic (indoor, outdoor settings) and real-world outdoor hazy images.

Figure 7 presents some dehazing outcomes from the Reside dataset (SOTS) on synthetic hazy images. As illustrated in Figure 7b,c, the methods show good performance in haze removal, however, TA-3DP [36] method’s shows some color distortion in the outputs. Also, MB-TF [37] produces over-saturated colors. Figure 7d method’s shows some residual haze.

In contrast, learning-based methods perform well in haze removal in most cases. However, they show some color distortions and poor saturation. For example, in Figure 7e CMTnet [15] results are substantially obscure in most cases (trees in the fourth image). As depicted from Figure 7f–h the results of GEN-ADV [38], DP-IPN [25], ADE-CGAN [39] have higher illumination, and some color shifts. Overall, as shown through all results in Figure 7, unlike our proposed model that can recover the real scene while preserving colours and saturation, most state-of-the-art compared methods fail to restore the hazy images and are unable to preserve colours and saturation.

Figure 8 shows some examples of haze removal results on three real-world challenging datasets: Dense-Haze (dense haze), NH-HAZE (non-homogonous haze), and O-HAZE (outdoor hazy images). At first glance, our results are visually almost identical to the ground-truth images. Contrary, as displayed in Figure 8b,c the methods TA-3DP [36], MB-TF [37] perform well in haze removal from dense-haze in most cases images and non-homogonous. Also Figure 8d GRIDdehaze-Net [26] method’s shows some light haze for example, the third and fourth images.

As shown in Figure 8e–h CMTnet [15], GEN-ADV [38], DP-IPN [25], ADE-CGAN [39] can successfully remove haze for most images, and the results are close to the ground-truth images to some extent. However, we can notice some color degradations for example, herbs in the second image tend to be dark green unlike in the ground truth which is light green. Also, these methods are unable to preserve edges and fine details, for example, the house cone in the first image. Additionally, all comparing methods fail to handle images with white objects or background (last image Figure 8).

To further assess the performance of our proposed approach, we carry out dehazig results of the comparing methods, including ours on real-world hazy images(without ground truth images), these images can be considered as challenging conditions hazy images (images with sky area, images with non-homogonous haze, images with low light condition). As demonstrated in Figure 9b,f,g TA-3DP [36], GEN-ADV [38], and DP-IPN [25] method’s can remove most of haze fromhazy images, however, the outputs have some color cost in sky area. Figure 9h shows ADE-CGAN [39] method’s results, it can be good in haze removal but it produces over-saturated colors. Alghough most of the comparing methods can remove haze to some extent, however, they have severe colour degradation in many scene parts, especially in the sky area (red frame shows the colour distortions and show some detail loss in different parts).

Differently, our proposed system can perform well in haze removal with colour and detail preserving. Overall, the proposed method can outperform the comparing methods in most cases, and has a superior generalization ability.

To validate all these qualitative evaluations, we conduct a quantitative comparison using FR-IQA (Full-reference Image Quality Assessement)and NR-IQA (no-reference Image Quality Assessement) in the next section.

#### 4.2.2. Quantitative Comparison on Both Real-World and Synthetic Hazy Images

In this section, we perform a quantitative comparison to rank the comparing methods, including ours. As we mentioned before, in our study we selected hazy images from a synthetic dataset Reside(SOTS, HSTS) and the three challenging real-world benchmarks (O-hazy, NH-hazy, Dense-hazy). Table 1 shows the average values of SSIM, PSNR and $\Delta E_{ab}^{*}$ indicators, where a higher value indicates the best one for all metrics.

By analyzing Table 1 SSIM, PSNR and $\Delta E_{ab}^{*}$ values, our proposed model achieved the best values for all SSIM, PSNR and $\Delta E_{ab}^{*}$ . This achievement indicates that our proposed method outperforms state-of-the-art dehazing methods in terms of structural perception and color preserving.

Additionally, we assess the accuracy of the restored images on both real-world and synthetic images by using some common *NR-IQA* (*FADE* [41], *e*, $\bar{r}$ ). The indexes *e* and $\bar{r}$ are two contrast enhancement indicators presented by Hautiere et al. [42], which measures the ability to recover the invisible edges and the quality of the contrast enhancement, respectively. *FADE* indicator evaluates the ability to remove haze from hazy images. Table 2 shows the average scores of *e*, $\bar{r}$ , *FADE*, and running time (T) of the comparative methods, including ours.

According to Table 2, our proposed model achieves the best scores (Values writen in bold on the table) for most of comparing metrics and ranks the second in recovering the visibility of edges.

In summary, both quantitative and qualitative analyses verify the effectiveness and the outstanding performance of our proposed attentional dehazing model, in terms of haze removal, color preserving, contrast enhancement, edges visibility and time complexity.

### 4.3. Ablation Studies

To validate the significance of the proposed attention module to enhance the performance of our dehazing model, we investigate two ablation studies with and without the attention module, as well as RGB-based model and RGB-HSV based model. Table 3 shows the SSIM and PSNR values of testing the dehazing model with and without the attention module with synthetic and both synthetic and real-world datasets. Table 4 shows the SSIM, PSNR and $\Delta E_{ab}^{*}$ values of testing the dehazing model in these cases.

As demonstrated from Table 3, our attention module has an outstanding ability to increase the performance of the dehazing process (SSIM and PSNR average values are the best in case of using our attention module). Additionally, employing both synthetic and real-world datasets has an excellent impact in enhancing dehazing performance (As marked in bold).

By contrast, the employing the integration of both RGB and HSV color space to implement our dehazing network contributes in enhancing color visibility and clarity while removing haze, Table 4 shows average values of SSIM, PSNR, and $\Delta E_{ab}^{*}$ are the best in case of RGB-HSV with both synthetic and real-world datasets.

## 5. Disscussion and Failure Cases

Based on our comprehensive analysis, the proposed method demonstrates superior performance compared to most state-of-the-art dehazing approaches, excelling in terms of haze removal, visibility enhancement, edge preservation, color preserving, and time complexity. Our method effectively addresses and overcomes the limitations observed in existing approaches.

The outstanding success of our proposed method is underscored by its robustness, efficacy, and efficient time complexity, achieved through the strategic utilization of both RGB and HSV color spaces, as well as the incorporation of the proposed attention module. These combined strengths significantly contribute to the exceptional results attained by our approach.

Combining RGB and HSV color spaces with an attention module can lead to a more comprehensive and adaptive approach for haze removal. This significant combination enables the algorithm to better understand and address the complexities of haze in diverse scenarios, contributing to more effective and robust results.

As any proposed network, our proposed network ${\mathrm{EIDC}}^{2}$ - Net has a failure case, ${\mathrm{EIDC}}^{2}$ - Net fails to handling super-resolution hazy image. This failure may stem from the inherent challenges associated with reconciling the intricate details required for super-resolution with the complexities introduced by atmospheric haze. The challenge lies in developing a sophisticated algorithm that can navigate this trade-off without compromising the effectiveness of either objective.

## 6. Conclusions

In this paper, we presented an innovative two-color space attention-based dehazing model ( ${\mathrm{ADMC}}^{2}$ -net), employing both HSV color space and RGB color space. This model structure incorporates two parallel densely connected sub-networks with an efficient fused channel-pixel attention module. This integration can solve several image dehazing issues (generalization, color degradation, and loss of fine details problems). Through extensive qualitative and quantitative comparisons and by analyzing some image quality metrics (SSIM, PSNR, $\Delta E_{ab}^{*}$ , e, r, FADE), our proposed model shows outstanding dehazing results that can outperform state-of-the-art dehazing methods in terms of haze removability, color preserving, time consumming, by solving most existing image dehazing limitations and treating all haze and light scene conditions. As a future work, we would investigate and incorporate advanced super-resolution techniques that can effectively handle hazy conditions. This may involve exploring deep learning architectures specifically designed for super-resolution in challenging environments.

## Figures and Tables

**Table 1:** Average Values of SSIM, PSNR and $\Delta E_{ab}^{*}$ of Comparing Methods on Synthetic and Real-world Datasets.

Dataset	Indicators	TA-3DP	MB-TF	GRIDdehaze	CMTnet	GEN-ADV	DP-IPN	ADE-CGAN	Ours
		[36]	[37]	[26]	[15]	[38]	[25]	[39]	
SOTS	SSIM	0.8239	0.9032	0.7242	0.8123	0.8247	0.9270	0.8003	0.9372
	PSNR	15.4630	19.2631	17.3572	18.0378	20. 3083	21.0383	19.0114	23.8275
	$\Delta E_{ab}^{*}$	0.8264	0.8104	0.8192	0.8634	0.7945	0.8184	0.8400	0.9517
HSTS	SSIM	0.7503	0.8505	0.7314	0.7502	0.7986	0.8732	0.8402	0.8987
	PSNR	15.0481	20.0072	16.8375	19.5837	17.5237	21.9170	18.4921	25.9730
	$\Delta E_{ab}^{*}$	0.8390	0.8700	0.8058	0.8227	0.8154	0.8048	0.7412	0.9301
O-hazy	SSIM	0.8817	0.9164	0.8901	0.8304	0.7509	0.9208	0.7979	0.9327
	PSNR	17.4005	20.1562	16.3421	17.4735	16.3782	19.0026	16.3420	22.7038
	$\Delta E_{ab}^{*}$	0.8002	0.8264	0.7401	0.8542	0.6834	0.8735	0.8830	0.9607
NH-hazy	SSIM	0.8302	0.8902	0.8892	0.8421	0.7304	0.8940	0.7380	0.9100
	PSNR	16.3971	25.0593	17.4217	16.8342	17.3179	22.9750	17.4400	27.3074
	$\Delta E_{ab}^{*}$	0.7824	0.7660	0.7391	0.8167	0.7940	0.7475	0.8927	0.9364
Dense-hazy	SSIM	0.7392	0.9472	0.7973	0.7321	0.8347	0.9150	0.8918	0.9830
	PSNR	18.6310	23.5809	16.5837	15.3791	18.9234	18.6832	18.3375	25.8373
	$\Delta E_{ab}^{*}$	0.7900	0.7245	0.7691	0.7632	0.8264	0.7820	0.8955	0.9402

**Table 2:** Average Values of FADE, e, r, and T(x) of Comparing Methods on Synthetic and Real-world Datasets.

Indicators	TA-3DP	MB-TF	GRIDdehaze	CMTnet	GEN-ADV	DP-IPN	ADE-CGAN	Ours
	[36]	[37]	[26]	[15]	[38]	[25]	[39]	
Fade	2.9201	1.9036	3.9478	2.8002	4.0084	3.8103	1.9387	**4.9036**
r	2.7297	**3.1207**	1.9824	1.8074	1.5982	2.0791	2.8897	2.9727
e	3.9023	2.3978	1.8079	2.8671	2.0072	1.9783	3.1207	**4.9802**
T(x)	0.4089	1.0082	0.3002	0.8902	0.4628	0.4001	0.3903	**0.3120**

**Table 3:** Average Values of SSIM and PSNR.

		Without Attention Module	With Attention Module
SSIM	T ∖ w S-H dataset	0.8097	0.9140
	T ∖ w S and R datasets	0.8257	**0.9472**
PSNR	T ∖ w S-H dataset	19.6783	22.8395
	T ∖ w S and R datasets	20.7103	**25.8016**

**Table 4:** Average Values of SSIM, PSNR and $\Delta E_{ab}^{*}$ .

		RGB	RGB-HSV
SSIM	T ∖ w S-H dataset	0.8997	0.9140
	T ∖ w S and R datasets	0.9035	**0.9631**
PSNR	T ∖ w S-H dataset	17.6021	19.0042
	T ∖ w S and R datasets	18.6453	**23.7341**
$\Delta E_{ab}^{*}$	T ∖ w S-H dataset	0.8541	0.9237
	T ∖ w S and R datasets	20.7103	**0.9741**

## Data Availability

Data are contained within the article.
